# Case Report: COVID-19-associated gangrene of fingers in a patient with autoimmune haemolytic anaemia

**DOI:** 10.3389/fmed.2026.1816083

**Published:** 2026-04-09

**Authors:** Mahmoud M. Ramadan, Mohammed Elmahal, Moustafa M. Madkour, Mohamed A. Eladl, Abdelraouf M. Abdelkarim, Zaid M. Abdelkarim, Mohammed A. Al-Shura, Wael A. Hassan, Ahmed Lamey

**Affiliations:** 1 Department of Clinical Sciences, College of Medicine, University of Sharjah, Sharjah, United Arab Emirates; 2 Department of Cardiology, Faculty of Medicine, Mansoura University, Mansoura, Egypt; 3 Department of Diabetes and Endocrinology, Queen Mary University of London, London, United Kingdom; 4 College of Pharmacy, University of Sharjah, Sharjah, United Arab Emirates; 5 Department of Basic Sciences, College of Medicine, University of Sharjah, Sharjah, United Arab Emirates; 6 Department of General Surgery, Faculty of Medicine, Kafrelsheikh University, Kafrelsheikh, Egypt

**Keywords:** autoimmune haemolytic anaemia, case report, COVID-19, gangrene, pulmonary embolism, digital ischaemia, immunothrombosis, arterial thrombosis

## Abstract

**Introduction:**

This case report describes a 50-year-old man with a 12-year history of long-standing warm-antibody autoimmune haemolytic anaemia (AIHA) who developed peripheral digital gangrene following COVID-19 infection. Although COVID-19-associated AIHA and COVID-19-associated digital ischaemia have each been reported separately, this case is notable for their coexistence with both venous and arterial thrombosis, resulting in tissue loss.

**Main symptoms and important clinical findings:**

The patient presented with a 3-day history of fever, cough, and shortness of breath. On examination, his temperature was 39.2 °C, radial pulse 120/min and regular, respiratory rate 25 breaths/min, blood pressure 148/88 mmHg, and oxygen saturation 82% on room air. Chest examination revealed bilateral basal and mid-zone crepitations.

**Main diagnoses, therapeutic interventions, and outcomes:**

The patient tested positive for COVID-19. Electrocardiography and echocardiography results were unremarkable. Laboratory investigations revealed severe haemolytic anaemia with a hemoglobin level of 5.7 g/dL and critical thrombocytopenia with a platelet count of 23 × 10^3^/μL. High-resolution computed tomography (CT) of the chest revealed bilateral multilobar ground-glass opacities with subpleural atelectatic bands, interlobular septal thickening, and a crazy-paving pattern, consistent with COVID-19 pneumonia. He was treated with non-invasive ventilation, corticosteroids, intravenous immunoglobulin, therapeutic anticoagulants, insulin glargine, and moxifloxacin. During admission, he received 6 units of packed RBCs and 34 units of platelets. His respiratory condition improved after 1 week, and therefore non-invasive ventilation was discontinued and he became stable on room air, with modest improvement in hemoglobin and platelet counts. Follow-up CT pulmonary angiography demonstrated a bilateral peripheral pulmonary embolism with regressing COVID-19 pneumonia. CT angiography of the upper extremities showed complete cessation of radial artery flow at both wrists. The patient subsequently developed dry gangrene affecting the right thumb and index finger and the distal parts of the left thumb and index finger. Six weeks post-admission, he underwent surgical amputation of the gangrenous digits.

**Conclusion:**

This case highlights a rare but serious coexistence of long-standing warm-antibody AIHA, COVID-19-associated thrombo-inflammatory disease, and combined venous and arterial thrombosis leading to peripheral digital gangrene. Although the exact mechanism cannot be established with certainty, the clinical course suggests that COVID-19 may have aggravated an already vulnerable haematologic and prothrombotic state, resulting in severe thrombotic complications and tissue loss.

## Background

Autoimmune haemolytic anaemia (AIHA) is an acquired heterogeneous autoimmune disorder characterized by the destruction of red blood cells (RBCs) owing to the production of autologous antibodies directed against erythrocyte antigens (1, 2). According to estimates, there are 1 to 3 occurrences per 100,000 people per year, making AIHA a relatively uncommon condition (1). Owing to this rarity, AIHA treatment is mostly based on results from limited prospective studies, case series, and empirical findings. Although most patients respond positively to steroids, relapses can occur, and maintenance dosages of steroids are usually required. Splenectomy is a useful second-line treatment that can result in long-term remission without further pharmacological intervention. Notably, rituximab is an effective treatment option for steroid-refractory patients, those who require large maintenance doses of steroids, and those who are not candidates for surgery (3).

Coronavirus disease 2019 (COVID-19), which is caused by severe acute respiratory syndrome coronavirus 2 (SARS-CoV-2), emerged in late 2019 and rapidly evolved into a global pandemic that challenged healthcare systems and clinicians worldwide. As the infection spread across continents, clinicians encountered a broad and evolving spectrum of clinical manifestations and complications. Although the initial focus was on respiratory symptoms and acute respiratory distress, it soon became clear that COVID-19 infection and complications extended far beyond the respiratory system (4, 5).

COVID-associated AIHA has been thoroughly documented in the literature, including in a recent systematic review of reported cases (6). Likewise, peripheral arterial complications, digital ischaemia, and gangrene are recognized vascular manifestations of COVID-19 (7). In addition, macrovascular thrombotic presentations involving digital ischaemia have been described, including a reported case of multiple strokes with digital ischaemia caused by brachial-circulation thrombosis associated with COVID-19 (8).

Here we describe a case of COVID-19-associated digital gangrene occurring in a patient with long-standing warm-antibody AIHA alongside pulmonary embolism with distal upper-limb arterial-flow cessation. The relevance and novelty of this report lie not in any particular manifestation alone, both of which have been described previously for COVID-19 patients, but rather in their coexistence with mixed macrovascular thrombosis and tissue loss in the same patient.

## Case report

This is a case of a 50-year-old man with known AIHA, warm-antibody type, diagnosed in 2008 based on a positive result for the direct antiglobulin (Coombs) test, high reticulocyte count, negative sickling test, normal G6PD activity, and normal bone-marrow biopsy. He was under maintenance therapy for AIHA and type 2 diabetes with the following drugs: prednisolone 5 mg OD (once daily), folic acid 5 mg OD, and metformin 500 mg BD (twice daily). He had no history of cardiovascular disease. He did not drink alcohol and was a non-smoker.

On 18 May 2020, he was brought by ambulance to the emergency department with a 3-day history of fever, breathlessness, and cough. The patient was concerned about having COVID-19, which might be fatal for him. Upon hospital admission, he was alert and oriented but experiencing severe respiratory distress, with an oxygen saturation of 82% on room air. Consequently, he was initiated on a non-rebreather oxygen mask at a flow rate of 15 L/min (FiO_2_ of 40%). However, the patient experienced intermittent desaturation.

A subsequent shift to BiPAP non-invasive ventilation (FiO_2_ 45%) resulted in improvement, maintaining an oxygen saturation of 96% with normalization of his arterial blood gases. An arterial line was inserted on the left side to facilitate repeated arterial blood-gas analysis. On physical examination, his tympanic temperature was 39.2 °C, radial pulse rate 120/min (regular, full, and equal on both sides), respiratory rate 25 breaths/min, and blood pressure 148/88 mmHg. Lung auscultation revealed bilateral basal and mid-zone crepitations. Apart from tachycardia, the cardiac examination was normal as were the abdominal, neurologic, and lower-limb examinations. Electrocardiography and echocardiography were both normal. The patient tested positive for COVID-19.

**Figure 1:**
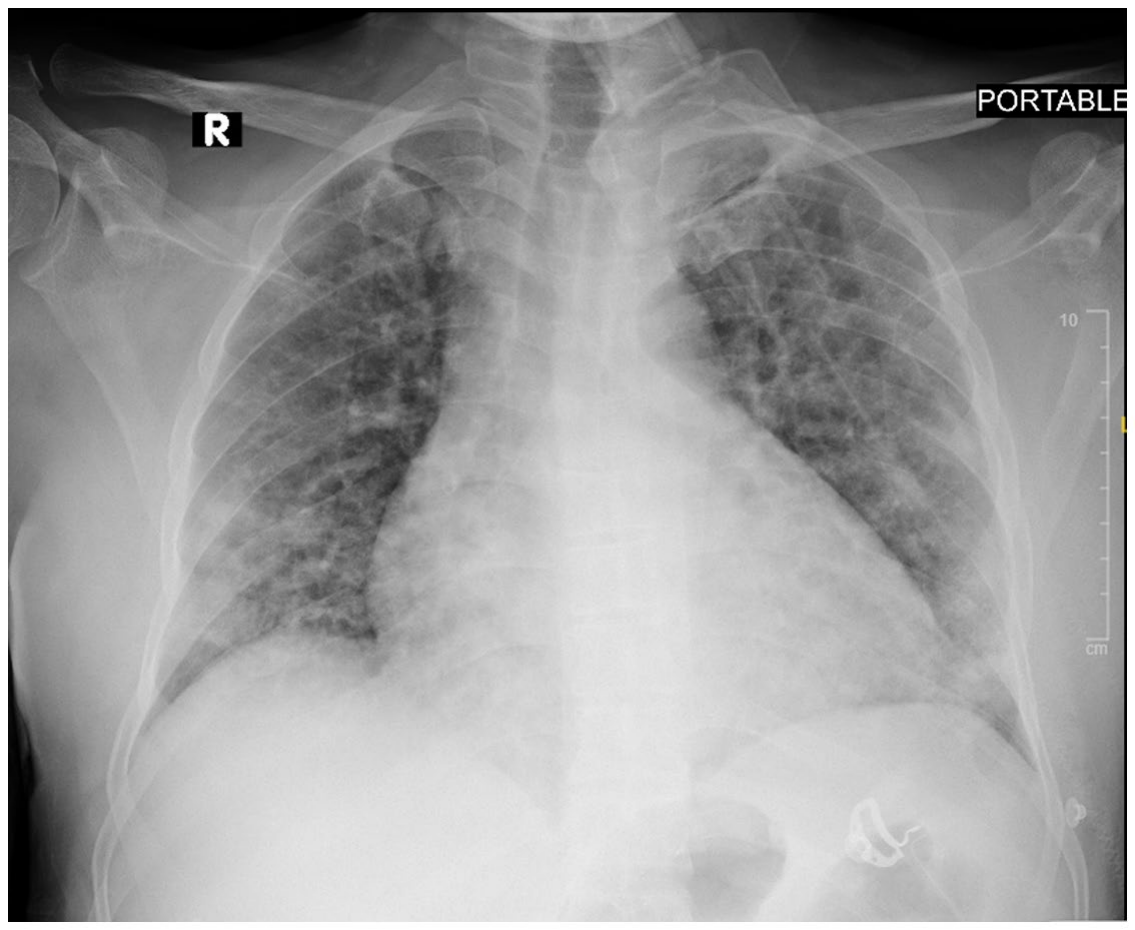
Portable anteroposterior chest radiograph showing diffuse bilateral peripheral patchy ground-glass opacities consistent with viral pneumonitis.

**Figure 2:**
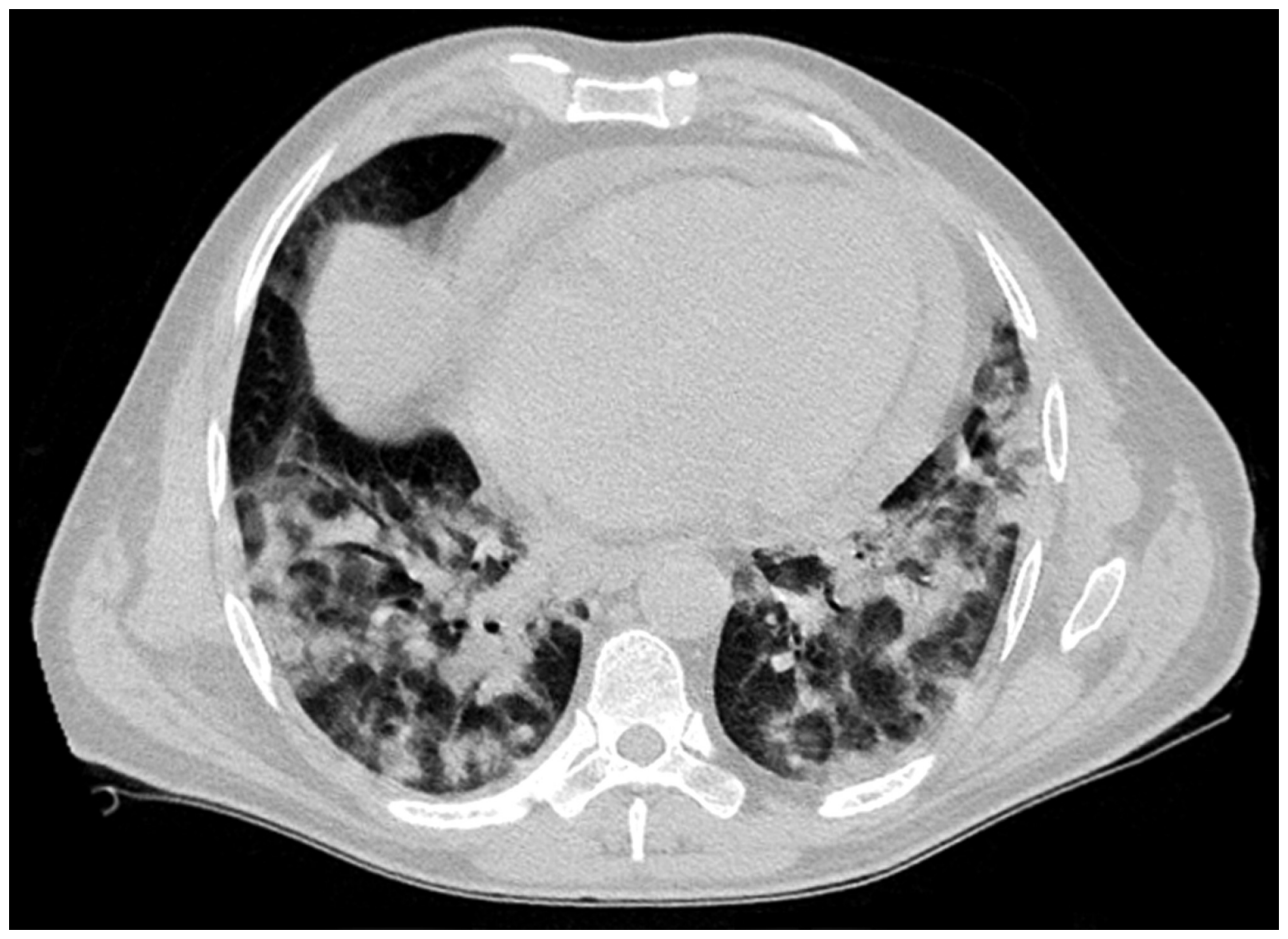
High-resolution computed tomography of the chest showing moderate pericardial effusion obliterating the anterior, posterior, and lateral recesses. The parenchyma of both lungs shows multi-lobar ground-glass opacities with no preferential predominance, superimposed with sub-pleural bands of atelectasis, interlobular septal thickening, and crazy-paving pattern typical of COVID-19 pneumonia.

Upon admission to the hospital, the patient was suffering from severe immune haemolytic anaemia, and results of tests given after admission revealed a marked drop of hemoglobin level down to 5.7 g/dL and critical thrombocytopenia with a total platelet count of 23 × 10^3^/μL (Table 1). Immediately, the patient was transfused with 2 units of packed red cells and 8 units of platelets. After the transfusion with blood products, he was feeling better and less irritable. A portable chest X-ray showed diffuse bilateral peripheral patchy ground-glass opacities suggestive of viral pneumonitis (Figure 1). High-resolution computed tomography (CT) of the chest showed moderate pericardial effusion obliterating the anterior, posterior, and lateral recesses, and both lung parenchyma showed multi-lobal ground-glass opacities with no preferential predominance superimposed with sub-pleural bands of atelectasis, interlobular septal thickening, and crazy-paving pattern compatible with COVID-19 pneumonia (Figure 2).

**Table 1:** Laboratory test results on admission and follow-up.

Laboratory test	Admission	2 weeks post-admission	3 weeks post-admission	6 weeks post-admission	Normal range
White blood cell indices					
Total WBC (×10^3^/μL)	12.23	16.19	15.99	9.17	3.5–10.5
Neutrophils (N/μL, %)	8,890 (72.7)	11,988 (74)	11,116 (69.5)	6,872 (74.9)	1,800–7,000
Lymphocytes (N/μL, %)	1,720 (14.1)	2,376 (14.7)	3,340 (21)	1,732 (18.9)	900–2,900
Monocytes (N/μL, %)	1,370 (11.2)	1,543 (9.5)	1,580 (9.9)	498 (5.4)	300–900
Eosinophils (N/μL, %)	10 (0.1)	95 (0.6)	70 (0.4)	43 (0.7)	100–500
Basophils (N/μL, %)	250 (2)	188 (1.2)	40 (0.3)	5 (0.05)	0–150
Red blood cell indices					
RBC (×10^6^/μL)	2.01	3.23	3.09	4.24	4.32–5.72
Hemoglobin (g/dL)	5.7	8.9	8.4	12	13–17
Hematocrit (%)	17.7	28.9	27.4	38.5	38.8–50
MCV (fL)	88.1	89.5	88.7	90.1	80–100
MCH (pg)	28.4	27.6	27.2	26.7	27–33
MCHC (g/dL)	32.2	30.8	30.7	29.6	32–36
RDW (%)	16.4	19.4	19	14.9	11.8–15.6
Reticulocyte count (%, number ×10^3^/μL)	2.12 (49)	–	–	–	0.5–2.5%
Platelet indices					
Platelet (×10^3^/μL)	23	82	104	165	150–450
MPV (fL)	11.7	11.9	11.4	11.5	6.5–12
PDW (fL)	18	19	17	15	9–16
Kidney function/electrolytes					
Sodium (mmol/L)	138	132	137	139	135–145
Potassium (mmol/L)	4.91	5.9	3.91	4.1	3.1–5.1
Chloride (mmol/L)	103	96	99	102	98–107
Calcium (mmol/L)	1.86	1.97	2.5	2.44	2.2–2.7
Phosphorus (mmol/L)	0.94	1.12	1.24	1.32	1.12–1.45
Magnesium (mmol/L)	0.91	0.86	0.97	0.95	0.65–1.05
Urea (mmol/L)	6.5	4.70	3.5	4.6	2.5–6.4
Creatinine (μmol/L)	82	59	56	73	62–115
Uric acid (μmol/L)	148	141	150	155	208–428
eGFR (mL/min/1.73 m^2^)	97.2	112.8	115.2	125	>90
Anion gap (mmol/L)	7	9	5	13	8–16
Iron studies					
Ferritin (μg/L)	4,210	1,540	693	236	24–336
Iron (μmol/L)	6.6	6.5	6.4	17.6	10–30
Liver function tests					
Total protein (g/L)	64	63	66	66	60–80
Albumin (g/L)	20.8	20	24	41	35–50
Total bilirubin (μmol/L)	31.6	22	8.3	11	2–17
Direct bilirubin (μmol/L)	10.2	9	5	4	0–6
Alanine aminotransferase (IU/L)	26	24	16	20	4–36
Aspartate aminotransferase (IU/L)	72	53	20	27	5–30
Alkaline phosphatase (IU/L)	82	111	55	65	30–120
Lactate dehydrogenase (IU/L)	797	897	816	110	50–150
Gamma-glutamyl transferase (IU/L)	63	44	32	21	5–40
Pancreatic enzymes					
Amylase (IU/L)	45	37	41	36	30–140
Lipase (IU/L)	86	65	77	48	<160
Cardiac enzymes					
Troponin-I (ng/L)	22	15	13	8	<40
CK-MB (IU/L)	11	6	8	11	5–25
Coagulation profile					
Prothrombin time (sec)	11.9	12	12	11.5	10–13
aPTT (sec)	56.3	31	30.9	30	21–35
INR	0.95	1.09	1.02	1.0	0.8–1.1
D-dimer (mg/L)	3.77	4.0	1.7	0.3	<0.55
Fibrinogen level (g/L)	0.5	5.5	4.3	3.3	2–4
Inflammatory markers					
C-reactive protein (mg/L)	36.8	53	47.5	7	8–10
Pro-calcitonin (μg/L)	1.35	0.04			<0.1
Others					
Random glucose (mmol/L)	13.4	10.5	8.2	7.2	4–11
Lactate (mmol/L)	1.1	–	–	–	<2
Vitamin D 25(OH) (nmol/L)	59.12	–	–	–	75–150
Osmolality (mOsm/Kg)	262	–	–	–	275–295
Cardiolipin IgG	Negative	–	–	–	–
Cardiolipin IgM	Negative	–	–	–	–
Arterial blood gases					
pH	7.44	–	–	–	7.35–7.45
paCO_2_ (mmHg)	38.3	–	–	–	35–45
paO_2_ (mmHg)	113.8	–	–	–	75–100
O_2_ saturation (%)	98	–	–	–	94–100
Total CO2 (mmol/L)	28.6	–	–	–	22–25
HCO_3_ (mEq/L)	25.8	–	–	–	22–26
FiO_2_ (%)	45	–	–	–	–

aPTT, activated partial thromboplastin time; eGFR, estimated glomerular filtration rate; FiO_2_, fraction of inspired oxygen; INR, international normalized ratio; MCH, mean corpuscular hemoglobin; MCHC, mean corpuscular hemoglobin concentration; MCV, mean corpuscular volume; MPV, mean platelet volume; PDW, platelet distribution width; RBC, red blood cells; WBC, white blood cells.

He was given oral prednisolone 40 mg OD, intravenous immunoglobulin, fondaparinux (factor Xa-inhibitor, an anticoagulant chemically related to low-molecular-weight heparin) 7.5 mg sc injection OD, ascorbic acid 1-g tablets OD, vitamin B complex with folic acid 5-mg tablets OD, pantoprazole 40-mg tablet OD, insulin glargine 12 units sc qPM, metformin 1-g tablets BD, sitagliptin 50-mg tablet BD, and moxifloxacin 400-mg tablet OD (for 2 weeks). Fondaparinux was selected as the initial anticoagulant because thrombosis risk was considered high despite thrombocytopenia, while heparin exposure was avoided in the setting of severe platelet reduction; this decision was individualized on the basis of overall thrombotic versus bleeding risk rather than a fixed platelet threshold. During anticoagulant therapy, the patient underwent daily clinical assessment for bleeding manifestations, with serial monitoring of hemoglobin, platelet count, and coagulation parameters including prothrombin time (PT), activated partial thromboplastin time (aPTT), and international normalized ratio (INR), and no clinically evident major bleeding occurred. The steroid (prednisolone) dose was increased from his chronic 5 mg OD maintenance regimen to 40 mg OD primarily because of acute haematologic deterioration, namely a presumed flare of warm-antibody AIHA with concomitant severe thrombocytopenia, rather than as a targeted treatment for COVID-19-associated inflammation alone.

At 3 days post-admission, his tympanic temperature dropped to 37.2 °C, monitored heart rate was 110/min with respiratory rate 30 breaths/min, blood pressure 125/77 mmHg, and oxygen saturation 95% on BiPAP non-invasive ventilation. He was alert and oriented but in marked distress with severe pallor. His hemoglobin level again dropped to 6.1 g/dL and his platelet count was just 32 × 10^3^/μL, due presumably to the continuous immune-mediated destruction of RBCs and platelets. Over the next 5 days, the patient received additional transfusions with 4 units of packed RBCs and 26 units of platelets along with methylprednisolone and intravenous immunoglobulin, which stabilized the hemoglobin level and platelet count (making a total of 6 units of packed RBCs and 34 units of platelets transfused since admission).

At 7 days post-admission, the patient’s general condition improved, the arterial cannula was removed, non-invasive ventilation was stopped, and the patient’s vital signs were stable, and he maintained a 96% oxygen saturation on room air. His temperature was 36.8 °C, heart rate 82/min, and blood pressure 125/68 mmHg. Lungs were clear to auscultation with non-labored respiration. Therefore, he was moved out of the critical care unit. His laboratory tests showed a modest improvement of hemoglobin level and red-cell indices and a modest increase of platelet count (to 82 × 10^3^/μL) compared to admission, but with rising total leucocytic count and neutrophil percentage. His D-dimer level was elevated (4 mg/dL) compared to the high baseline value at admission (3.77 mg/dL), and C-reactive protein level showed a rising titer (Table 1). A COVID-19 test report was negative after 3 nasopharyngeal swabs. The right index finger and thumb showed bluish discolouration with no blackening although his radial and brachial pulses were palpable with a normal volume. The left index finger was slightly bluish at the distal phalanx, but his left thumb had normal color, and both fingers were colder than the rest of the fingers.

**Figure 3:**
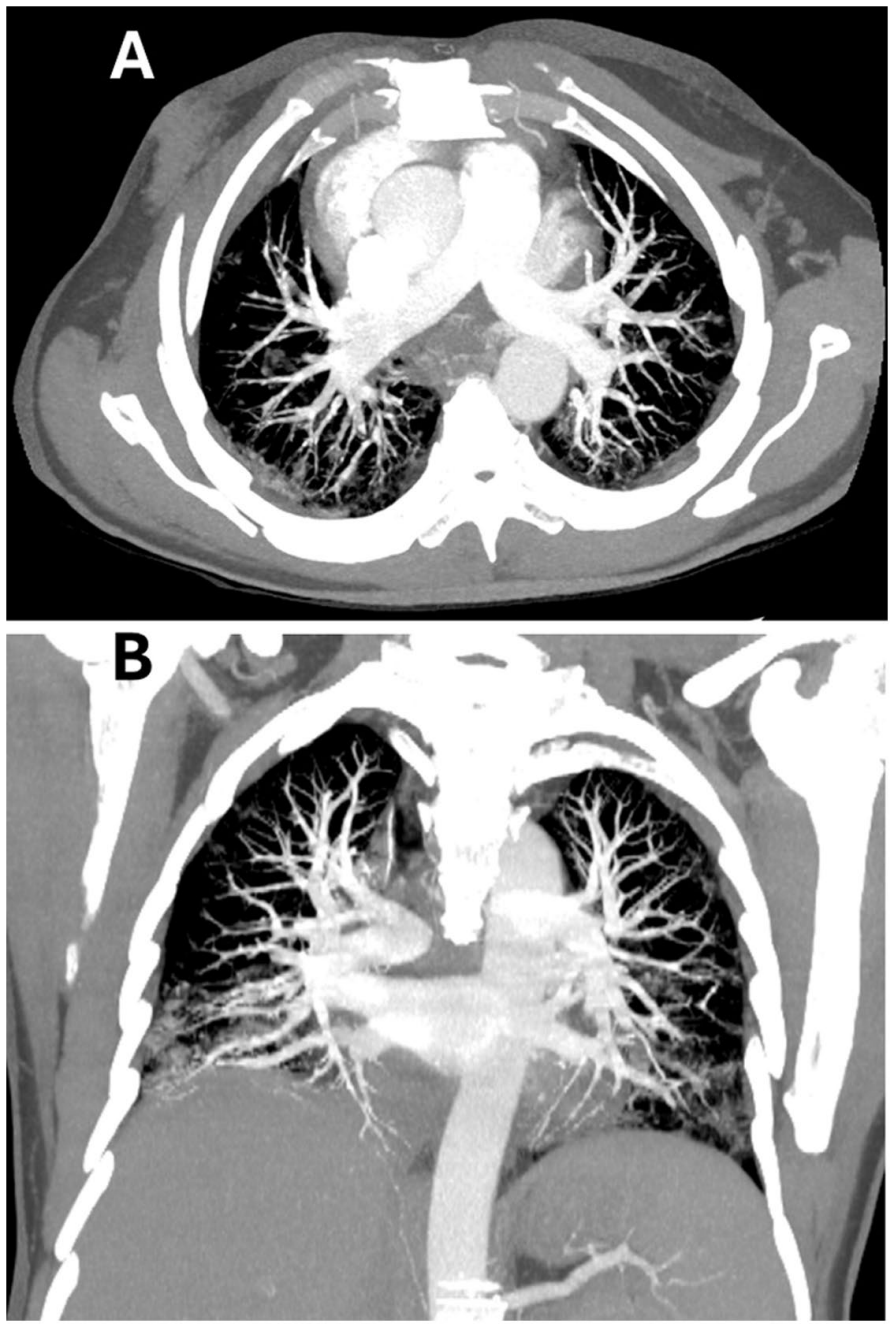
Axial **(A)** and coronal **(B)** computed tomography pulmonary angiogram images demonstrating bilateral peripheral pulmonary emboli with partial luminal occlusion. The posterior descending branches of both pulmonary arteries are seen partially obliterated by thrombotic material with normal contrast opacification of the distal segments denoting partial occlusion, with moderate pericardial effusion, and regressing bilateral interstitial COVID-19 pneumonia.

Seven days later (2 weeks post-admission), follow-up CT pulmonary angiography was performed and revealed a bilateral peripheral pulmonary embolism with partial luminal obliteration (the posterior descending branches of both pulmonary arteries were partially obliterated by thrombotic material with normal contrast opacification of the distal segments denoting partial occlusion), moderate pericardial effusion, and bilateral interstitial COVID-19 pneumonia, implying a regressive course compared to the prior scan (Figures 3A,B). However, the right thumb and index showed increased dark discolouration.

**Figure 4:**
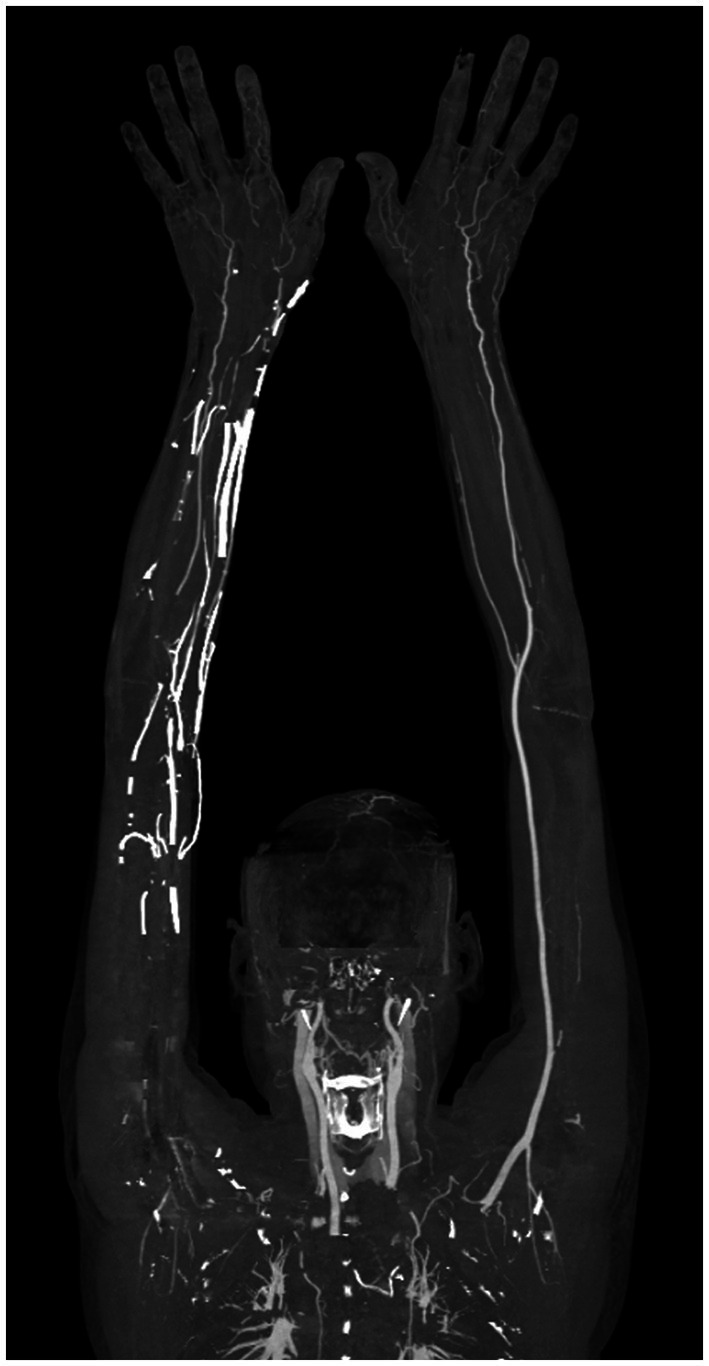
Computed tomography angiogram maximum-intensity projection of both upper limbs. This available archived image provided an overview of the upper-extremity arterial circulation. Although a discrete intraluminal thrombus or sharply defined contrast cutoff could not be optimally visualized on this single reconstruction, the image is concordant with the radiology-reported cessation of radial artery flow at the wrist bilaterally.

One week later (3 weeks post-admission), CT angiography of the upper limbs revealed cessation of radial artery flow at the wrist bilaterally, with both subclavian arteries being normal, normal opacification of both upper-limb arteries proximally (brachial and axillary) with neither stenosed nor occluded segments, and bilateral ulnar artery dominance (normal variant) with good opacification of both deep and superficial palmar arches and the digital arteries (Figure 4). However, only a single archived CT angiography reconstruction was available for inclusion in this report, which provided an overall angiographic overview but did not optimally delineate the exact level of occlusion.

After documenting the pulmonary embolism and distal upper-limb arterial thrombosis and with partial recovery of the platelet count compared with admission and no major bleeding, the vascular surgeon refrained from prescribing heparin because of thrombocytopenia concerns, and the patient was transitioned to apixaban 5 mg BD for ongoing anticoagulation.

**Figure 5:**
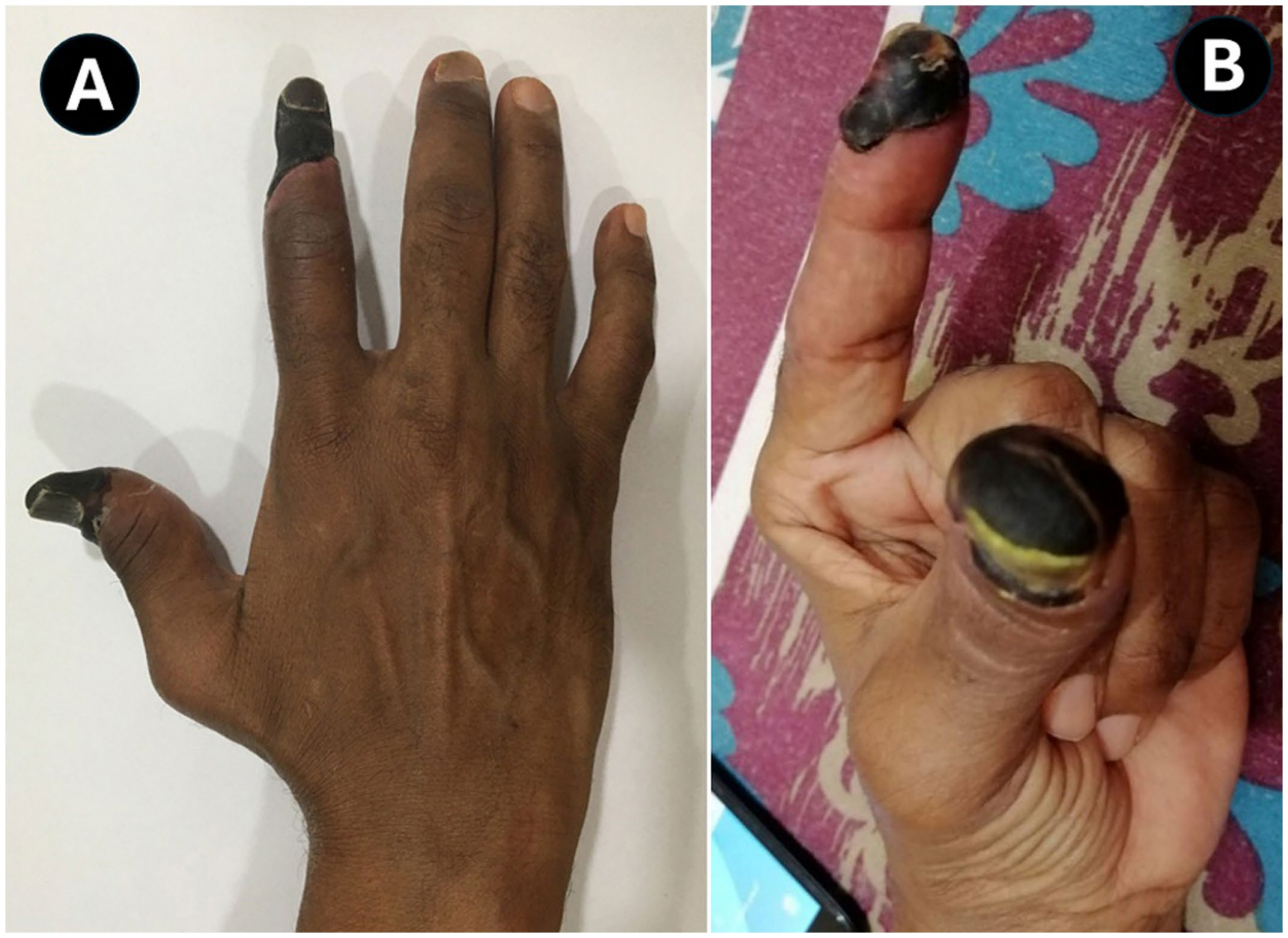
Dry gangrene of the right thumb and index finger **(A)** and acral gangrene involving the left thumb and index fingertip **(B)**.

At 4 weeks post-admission, the patient’s vital signs were stable with a temperature of 36.9 °C, heart rate 85/min, blood pressure 124/76 mmHg, and oxygen saturation 98% on room air, so that he was discharged to his home. He had a dry gangrene of the right index finger and thumb and the acral tips of the left index finger and thumb (Figure 5), although the radial and ulnar pulses could be felt distally. The patient had a low but reasonable platelet count of 98 × 10^3^/μL and hemoglobin level of 9.8 g/dL. No vascular intervention was deemed necessary, but the patient was advised to follow up with the vascular surgery clinic and keep the fingers dry and free of infection with daily wiping with an alcohol swab to aid in the demarcation. He was also advised to taper the prednisolone dose down to the 5 mg maintenance level that he had taken previously, monitor his CBC weekly, monitor blood sugar daily to achieve tight control in preparation for surgery (finger amputation), and continue apixaban 5 mg BD (and in case of bleeding to rush to the nearest hospital).

At 2 weeks post-discharge (6 weeks post-admission), the patient was readmitted for amputation of his gangrened fingers. He was ambulant, alert, oriented, and cooperative with stable vital signs and a normal temperature of 37 °C, heart rate 83/min (regular, full, and equal), respiratory rate 18 breaths/min, weight 75 kg, height 176 cm, BMI 25 kg/m^2^, oxygen saturation 98% on room air, and blood pressure 122/72 mmHg. Electrocardiography and echocardiography results were normal, and a chest X-ray showed right lower-zone minimal infiltrates with bilateral prominent broncho-vascular markings. The COVID-19 test was negative. His laboratory tests showed marked improvement of the hemoglobin level and RBC count, although still not fully normalized, with normalization of the total leucocytic count, platelet count, and C-reactive protein. His medications included amlodipine 5 mg OD, prednisolone 5-mg tablets OD, and oral hypoglycaemic drugs (metformin 1 g BD and sitagliptin 50 mg BD); apixaban was stopped. Subsequent surgery followed an uneventful course and comprised amputation of the distal phalanx of the right thumb and of the distal and middle phalanges of the right index finger as well as debridement of the acral tips of the left index finger and thumb. Apixaban was withheld before surgery and resumed post-operatively to complete a three-month therapeutic course, after which it was discontinued because the pulmonary embolism and distal arterial thrombosis were considered provoked in the setting of acute COVID-19-associated thrombo-inflammatory disease; no recurrent thrombotic or major bleeding events were reported during follow-up. The patient was stable on follow-up and returned to his work and usual life activities, with no other health events reported to date.

## Discussion

This case constitutes an example of uncommon multi-systemic manifestations associated with the novel coronavirus SARS-CoV-2. AIHA is a rare hematologic disorder characterized by the destruction of RBCs by the immune system, leading to haemolysis and potentially severe anaemia (9). COVID-19, caused by the SARS-CoV-2 virus, has been suggested to trigger AIHA in some cases, possibly due to immune dysregulation and the production of autoantibodies (10). It is essential to recognize this association, as it is challenging to diagnose hematologic and thromboembolic events in COVID-19 patients because such events can be masked by the features of COVID-19 itself. In this context, very few cases have been reported concerning AIHA being associated with or exacerbated by COVID-19 infection, and no cases have reported gangrene as a complication (11–16). Additionally, anaemia characterized by lower levels of hemoglobin on admission has been shown to be significantly associated with more severe COVID-19 infections as measured by longer hospital stays and relatively higher mortality (17).

To date, there has been a paucity of reports published that address the occurrence of dry gangrene in individuals diagnosed with COVID-19, and thus it is considered an extremely rare complication (18–21). However, peripheral gangrene in AIHA patients diagnosed with COVID-19 has never been reported. The patient described in the current case report had pre-existing AIHA and developed peripheral gangrene in his fingers after contracting COVID-19. Although the direct association between AIHA, COVID-19, and peripheral gangrene is not extensively documented in the literature, several interconnected factors may contribute to this phenomenon.

From a diagnostic perspective, the combination of severe anaemia, marked thrombocytopenia, elevated LDH, and thrombotic events should not be attributed to AIHA exacerbation alone. The coexistence of AIHA and thrombocytopenia may fit an Evans-syndrome phenotype (22), but it also warrants consideration of thrombotic microangiopathy (TMA), which is typically characterized by microangiopathic haemolytic anaemia, thrombocytopenia, and ischemic end-organ injury, with complement activation representing an important underlying mechanism in several TMA subtypes (23). Importantly, recent evidence indicates that COVID-19 can act as a trigger for TMA, including complement-mediated pathways, particularly in predisposed patients (24). In parallel, COVID-19-associated coagulopathy should be distinguished from overt disseminated intravascular coagulation (DIC), as these processes overlap clinically yet are not identical (25). In the present case, the previously established DAT (direct antiglobulin test)-positive warm-antibody AIHA supports immune haemolysis as a major contributor; however, because schistocyte assessment, ADAMTS13 activity, complement studies, and formal DIC scoring were not available, a concurrent TMA/complement-mediated microangiopathy or DIC could not be definitively excluded. We therefore interpret this case more cautiously as warm-antibody AIHA with concomitant thrombocytopenia complicated by COVID-19-associated immunothrombosis, while acknowledging the possibility of an overlapping microangiopathic process.

A further limitation of our assessment of this case is the absence of a peripheral blood smear and extended haemolysis/complement work-up, including haptoglobin, plasma-free hemoglobin, and complement studies, which would have helped distinguish isolated immune haemolysis from an overlapping microangiopathic or complement-mediated process. Although warm-antibody AIHA usually provokes reticulocytosis, the relatively modest admission reticulocyte response in this case may indicate inadequate bone marrow compensation, with possible ineffective erythropoiesis and/or inflammatory cytokine–mediated suppression contributing to the severity of anaemia (26).

Importantly, the coagulation profile of the patient upon admission—including markedly elevated D-dimer, prolonged aPTT, and profound hypofibrinogenaemia—is more suggestive of a consumptive coagulopathy/DIC-like state than of isolated hypercoagulability alone. The subsequent rise in fibrinogen may reflect recovery from early consumption together with a persistent acute-phase inflammatory response; accordingly, the thrombotic phenotype in this case is better interpreted as an overlap between COVID-19-associated immunothrombosis and probable consumptive coagulopathy (25).

Another limitation is that the thrombophilia assessment was incomplete: antibodies specific for cardiolipin were reported for our patient, but lupus anticoagulant and anti-β2-glycoprotein-I antibodies were not available, precluding a full evaluation of antiphospholipid syndrome. Broader thrombophilia testing could also be considered in such mixed arterial and venous thrombotic presentations, although its diagnostic yield in the acute phase of severe systemic illness may be limited (27).

Multiple mechanisms have been suggested to explain the pathogenesis of the procoagulant condition associated with AIHA, potentially heightening the risk of thrombosis and peripheral ischemia. The mechanisms of increased thrombosis in AIHA may involve RBC membrane destruction and release of cellular constituents (cell-free hemoglobin, erythrocyte arginase, free haem, and ADP), which in turn activate the coagulation cascade (28). This inherent propensity toward hypercoagulability becomes more precarious when coupled with the hypercoagulable state associated with COVID-19 (29), as there is a high incidence of thrombotic events and coagulation abnormalities in COVID-19 patients (30), which could further exacerbate the risk of peripheral gangrene.

Additionally, both AIHA and COVID-19 are known to promote endothelial dysfunction, potentially facilitating microvascular thrombosis and ischemic events (31, 32). Further support can be provided to this notion by the increased D-dimer and thrombocytopenia found in COVID-19 patients (33, 34). Moreover, the immune dysregulation characteristic of both AIHA and COVID-19, including a possible hyperinflammatory host response to SARS-CoV-2, may contribute to vascular inflammation and thrombosis, thereby adding to the risk profile for peripheral gangrene (35, 36). However, because IL-6, soluble IL-2 receptor, triglycerides, and related cytokine storm–associated biomarkers were not measured in this case, any suggestion of a true cytokine-storm syndrome remains hypothetical and should be interpreted cautiously.

SARS-CoV-2 may also infect and damage RBCs, leading to amplification of haemolytic and prothrombotic tendencies and potentially increasing the likelihood of gangrene development; although this mechanism is still debated and the evidence is limited and inconclusive (37, 38), exploring this possibility could shed light on its potential implications for individuals with AIHA and the development of gangrene after COVID-19 diagnosis.

A key strength of the assessment of this case is that the clinical course aligns with the current concept of COVID-19 as a vascular/endothelial disease. Endothelial activation or injury can shift the vascular surface toward a proadhesive and procoagulant phenotype, promoting platelet adhesion and localized thrombosis (39, 40). Autopsy and histopathologic studies have demonstrated endothelialitis, microvascular thrombosis, and angiopathic changes in COVID-19 patients, supporting a primary role for the endothelium in severe disease (41, 42).

In parallel, innate immune activation increases the potential for coagulation. Activation of both the complement system and neutrophils can induce the release of neutrophil extracellular traps, which provide a scaffold for platelet aggregation and fibrin deposition. Neutrophil extracellular traps may be enriched with tissue factor, directly coupling innate immune effector pathways to thrombin generation and clot propagation—one of the clearest mechanistic bridges between innate immunity and coagulation in COVID-19 (40). This integrated endothelium–innate immune–coagulation loop is widely referred to as immunothrombosis and explains why thrombosis can occur in both macro- and microvascular beds, including distal arteries, sometimes despite preserved proximal pulses (39–42).

A compelling question may arise: Why might AIHA intensify immunothrombosis in COVID-19 patients? In fact, AIHA itself carries an increased thrombotic risk, particularly during active haemolysis (2, 28). Haemolysis releases erythrocyte-derived mediators (e.g., cell-free hemoglobin, free haem, ADP, and procoagulant microparticles) that can impair nitric oxide bioavailability, promote endothelial dysfunction, and amplify coagulation signaling (28, 31). Therefore, our patient’s AIHA flare (marked anaemia and thrombocytopenia requiring extensive transfusion and immunosuppression) may have created a primed baseline state onto which COVID-19-driven immunothrombosis was superimposed (33, 40, 42). This convergence provides a biologically plausible explanation for the sequence of events observed for this patient: persistent inflammatory activation followed by pulmonary embolism and by distal upper-limb arterial-flow cessation, ultimately leading to progressive acral ischemia and gangrene (33, 43).

From a therapeutic perspective, escalation from chronic prednisolone 5 mg/day to prednisolone 40 mg/day and subsequently methylprednisolone was undertaken primarily to control the presumed AIHA flare with concomitant severe thrombocytopenia, given the marked anaemia, critical platelet fall, and ongoing transfusion requirement, rather than as specific anti-inflammatory therapy for COVID-19 alone. Because long-term prednisolone exposure at approximately 5 mg/day may suppress the hypothalamic–pituitary–adrenal axis, the possibility of adrenal suppression is also relevant in the clinical context. However, the patient did not manifest refractory hypotension or other features that are strongly suggestive of adrenal crisis, and the escalated glucocorticoid regimen itself would likely have provided supraphysiologic coverage during the acute illness.

Anticoagulation in this case required individualized balancing of thrombosis and bleeding risks. The patient initially presented with severe thrombocytopenia, which complicated therapeutic anticoagulation; however, the coexistence of severe COVID-19, active haemolysis, markedly elevated D-dimer, and subsequently confirmed pulmonary embolism and distal arterial occlusion indicated a substantial thrombotic burden. Fondaparinux was used initially to provide anticoagulant coverage while avoiding heparin exposure in the context of thrombocytopenia. Later, once thrombosis had been objectively documented and the platelet count had partially improved relative to admission without major bleeding, apixaban was used for ongoing management. This sequence reflects a pragmatic, case-specific approach to COVID-associated coagulopathy rather than reliance on a single platelet threshold.

As a clinical implication for physicians, the key lesson is vigilance for thrombotic complications (both venous and arterial) in COVID-19 patients with underlying autoimmune haematologic disorders, particularly when D-dimer and inflammatory markers remain elevated or when new acral discolouration appears. Early vascular imaging and multidisciplinary management (hematology, vascular surgery, critical care) are essential, while balancing bleeding risk in thrombocytopenic patients.

From the perspective of prevention, if a similar patient presented today, early antiviral therapy might be considered at the beginning of infection in eligible high-risk patients to reduce viral replication and possibly attenuate the downstream inflammatory and thrombotic cascade (40, 44). However, this patient was admitted on 18 May 2020, before the availability of current oral antiviral agents such as nirmatrelvir/ritonavir. In current practice, these oral antivirals are primarily used for early mild-to-moderate COVID-19 in non-hospitalized patients at risk of progression (44), whereas remdesivir has an approved role for hospitalized patients as well as selected high-risk non-hospitalized patients. Nevertheless, direct evidence that antiviral therapy specifically prevents acral ischemia or digital gangrene remains lacking. Therefore, prevention in similar cases is more likely to depend on early recognition of immunothrombosis complications, close monitoring of haemolysis and coagulation markers, prompt vascular imaging when acral discolouration appears, and timely multidisciplinary management including individualized antithrombotic therapy when clinically appropriate (33, 43).

## Conclusion

This case highlights an uncommon and clinically important coexistence of long-standing AIHA, COVID-19-associated thrombo-inflammatory disease, mixed macrovascular thrombosis, and peripheral digital gangrene. Although the exact mechanisms remain unclear, immune dysregulation, endothelial dysfunction, and direct viral effects are plausible contributors to AIHA in the context of COVID-19. Clinicians should remain vigilant for hematologic complications in COVID-19 patients with pre-existing hematologic disorders, and further research is needed to better understand these complex interactions.

## Data Availability

The raw data supporting the conclusions of this article will be made available by the authors, without undue reservation.
